# Supplementary material for: Mitochondrial oxidant stress promotes α-synuclein aggregation and spreading in mice with mutated glucocerebrosidase
Source: NPJ Parkinsons Dis. 2024 Dec 11;10:233. doi: 10.1038/s41531-024-00842-8 (PMC11634889; doi:10.1038/s41531-024-00842-8)
Supplement: Supplementary file 1 — Supplementary figures and table [file 41531_2024_842_MOESM1_ESM.pdf]

Supplementary Information

Mitochondrial oxidant stress promotes α-synuclein aggregation and spreading in mice with mutated glucocerebrosidase

Pietro La Vitola,[1,2] Eva M Szegö,[1,2] Rita Pinto-Costa,[1] Angela Rollar,[1,2] Eugenia Harbachova,[1,2] Anthony HV Schapira,[2,3] Ayse Ulusoy[1,2] and Donato A Di Monte[1,2]

[1] German Center for Neurodegenerative Diseases (DZNE), Bonn 53127, Germany

[2] Aligning Science Across Parkinson's (ASAP) Collaborative Research Network, Chevy Chase, MD 20815, USA

[3] Department of Clinical and Movement Neurosciences, University College London Queen Square Institute of Neurology, Royal Free Campus, London NW3 2PF, UK

Supplementary Figure 1

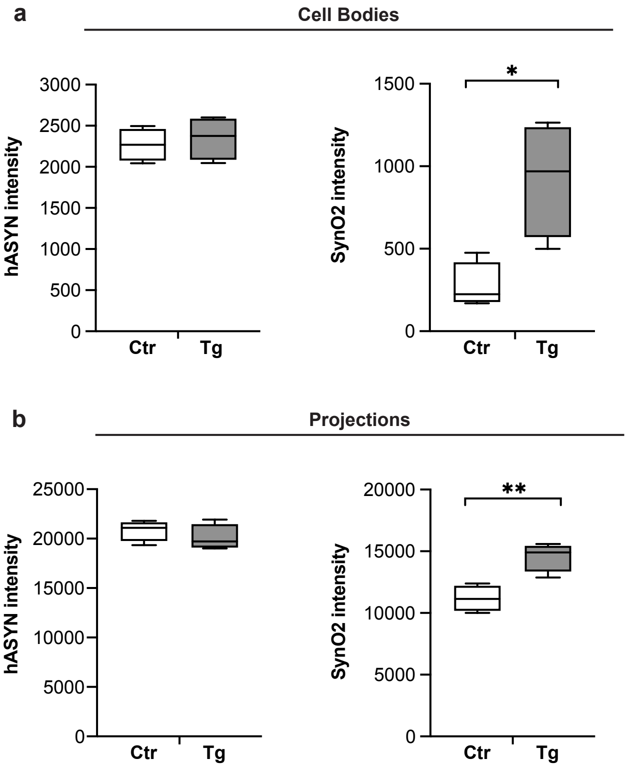

Supplementary Fig. 1 / L444P-induced ASYN aggregation assessed by SynO2 staining. All mice received an intravagal injection of hASYN-delivering AAVs. MO tissue sections were double-stained with anti-hASYN and an antibody, SynO2, that reacts with aggregated ASYN species. **a, b** Fluorescent intensity (expressed as arbitrary units) of hASYN and SynO2 signals were measured in hASYN-containing neuronal cell bodies (**a**) and projections (**b**) in the left (ipsilateral to the AAV injection) DMnX of control (Ctr) and L444P mutant (Tg) mice (n = 4/ group). Plots show median, upper and lower quartiles, and maximum and minimum as whiskers. *$p \leq 0.05$ and **$p \leq 0.001$, Student's t-test.

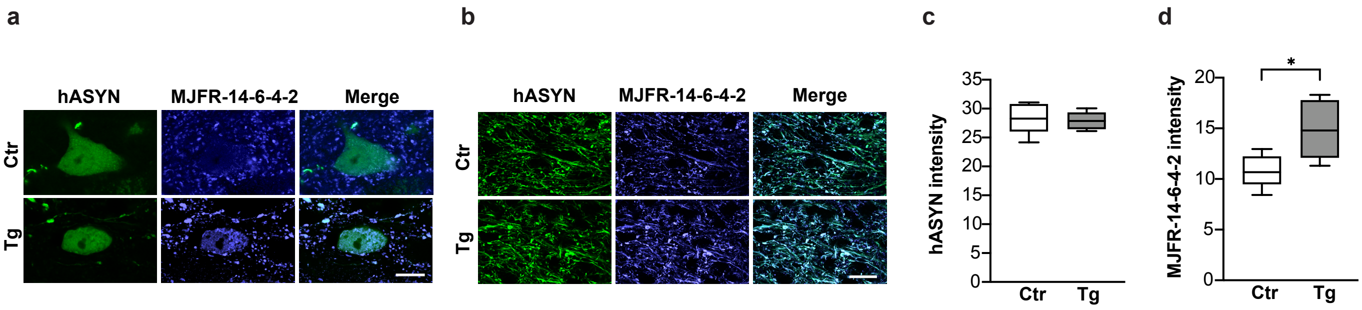

Supplementary Fig. 2 / L444P-induced ASYN aggregation assessed by MJFR-14-6-4-2 staining. All mice received an intravagal injection of hASYN-delivering AAVs. MO tissue sections were double-stained with anti-hASYN and an antibody, MJFF-14-6-4-2, that reacts with aggregated ASYN species. **a, b** Representative fluorescent images of neuronal cell bodies (**a**) and projections (**b**) in the left (ipsilateral to the AAV injection) DMnX of a control (Ctr) and an L444P mutant (Tg) mouse. Scale bar: 10 μm (**a**) and 20 μm (**b**). **c, d** Fluorescent intensity (expressed as arbitrary units) of hASYN immunoreactivity was measured in the left DMnX (**c**); fluorescent MJFF-14-6-4-2 intensity was measured within hASYN-containing DMnX neurons (both cell bodies and neurites) (**d**); samples were obtained from control and L444P mutant mice (n = 5/group). Plots show median, upper and lower quartiles, and maximum and minimum as whiskers. *$p \leq 0.05$, Student's t-test.

Supplementary Figure 3

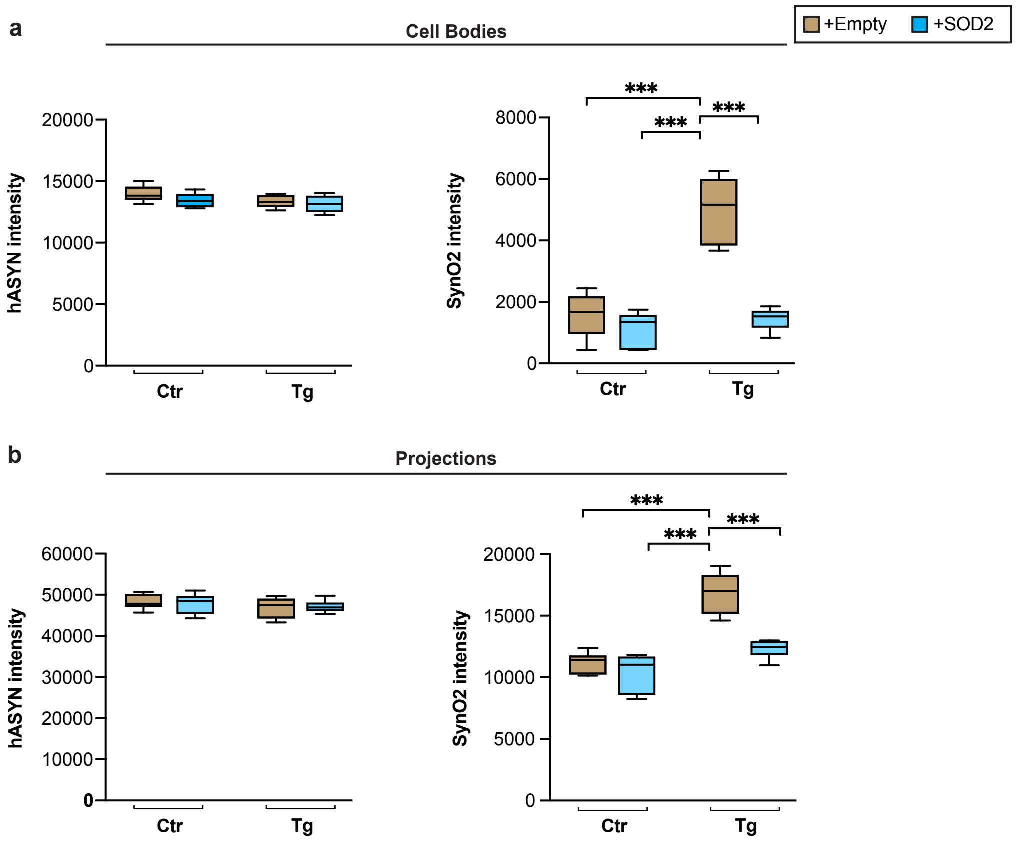

Supplementary Fig. 3 / Effects of SOD2 transduction on L444P-induced ASYN aggregation. Control (Ctr) and L444P mutant (Tg) mice were all injected intravagally with hASYN-delivering AAVs. Groups of animals also received, together with hASYN-AAVs, empty vectors lacking protein coding (+empty) or AAVs delivering SOD2 DNA (+SOD2). MO tissue sections were double-stained with anti-hASYN and anti-SynO2. **a, b** Fluorescent intensity (expressed as arbitrary units) of hASYN and SynO2 signals in hASYN-containing neuronal cell bodies (**a**) and projections (**b**) in the left DMnX of control and L444P mutant mice co-injected with either hASYN/empty- or hASYN/SOD2-AAVs ($n \geq 5$/group). Plots show median, upper and lower quartiles, and maximum and minimum as whiskers. * $p \leq 0.05$ and **$p \leq 0.001$, Tukey post-hoc test.

Supplementary Figure 4

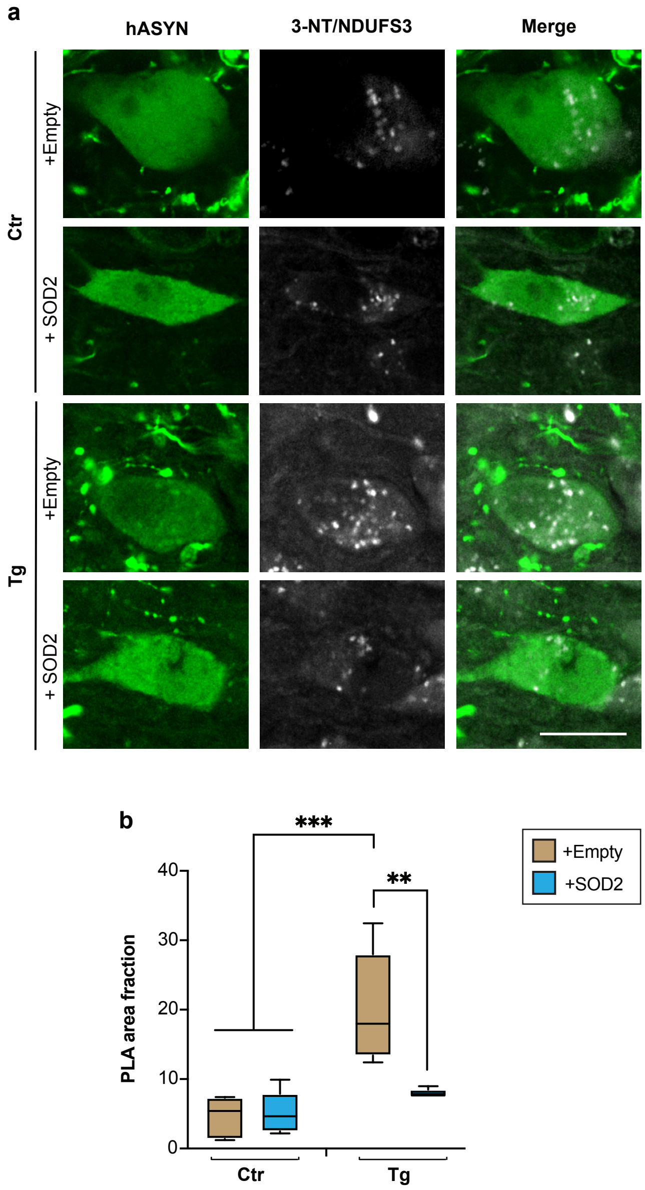

Supplementary Fig. 4 / L444P-induced nitration of the mitochondrial complex I subunit NDUFS3. All animals were injected intravagally with hASYN-delivering AAVs. Two groups of control (Ctr) mice also received either empty vectors lacking protein coding (+empty) or AAVs delivering SOD2 DNA (+SOD2). Similarly, two groups of L444P mutant (Tg) animals were injected intravagally with empty or SOD2-AAVs. MO sections were processed for PLA detecting nitrated NDUFS3 and then labelled with anti-hASYN. **a** Representative fluorescent images of neurons in the left (ipsilateral to the AAV injections) DMnX. Images show an overt increase in PLA signal in the sample from a transgenic mouse treated with hASYN/empty-AAVs. This increase did not occur in the DMnX of another L444P/wt animal injected with hASYN/SOD2-AAVs. Scale bar: 10 μm. **b** The PLA area fraction was quantified in fluorescent samples from Ctr and Tg mice co-injected with either hASYN/empty- or hASYN/SOD2-AAVs ($n \geq 5$/group). Plots show median, upper and lower quartiles, and maximum and minimum as whiskers. $^{**}p \leq 0.01$ and $^{***}p \leq 0.001$, 2-way ANOVA followed by Tukey's test.

Supplementary Figure 5

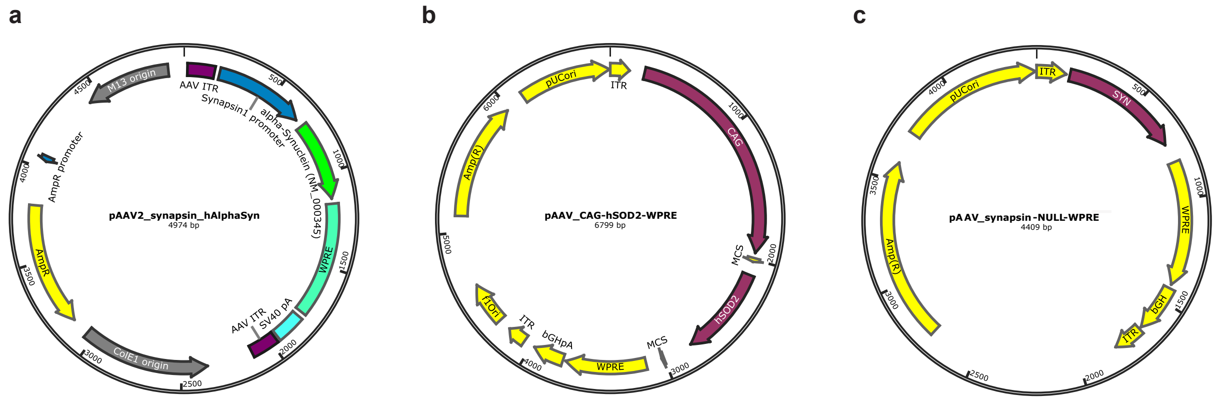

Supplementary Fig. 5 / Maps of AAVs used in the study. Maps showing the expression cassettes of vectors used for transduction of hASYN (**a**) or SOD2 (**b**). The map of empty AAVs lacking protein coding sequence is also shown in (**c**).

Supplementary Figure 6

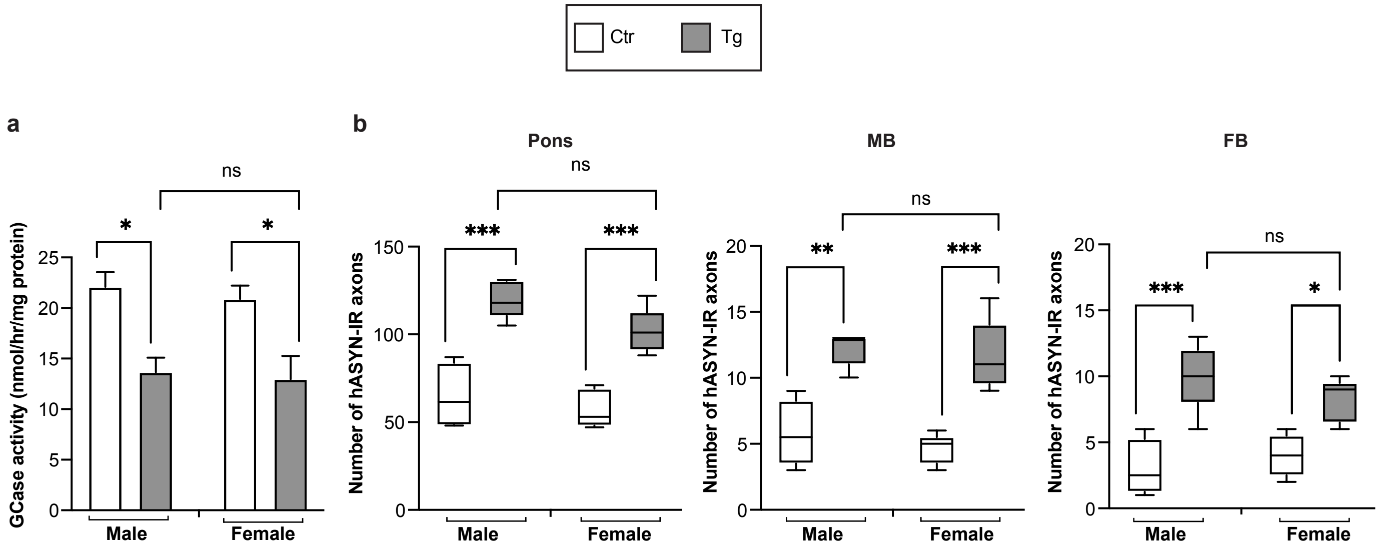

Supplementary Fig 6 / Lack of sex-dependent differences in L444P/wt mice. a GCase activity in whole brain homogenates from naïve (untreated) male and female control (Ctr, n = 3 males and 3 females) and L444P mutant (Tg, n = 4 males and 3 females) mice. A significant decrease in enzyme activity was associated to the mutated GCase. The extent of this effect was comparable in male and female L444P mutant animals (ns = not statistically significant). **b** Mice received an intravagal injection of hASYN-delivering AAVs. Tissue sections throughout the brain were stained with anti-hASYN. The number of hASYN-immunoreactive (IR) axons was counted in sections of the left pons, midbrain (MB) and forebrain (FB) from male and female control (n = 4 males and 5 females) and L444P mutant (n = 5 males and 5 females) mice. A significant increase in counts of hASYN-containing axons was consistently observed in transgenic mice. The extent of this effect was comparable in male and female L444P mutant animals. Plots show median, upper and lower quartiles, and maximum and minimum as whiskers. $^*p \leq 0.05$, $^{**}p \leq 0.01$ and $^{***}p \leq 0.001$, 2-way ANOVA followed by Tukey's test.

Supplementary Table 1 Source data for figures in the manuscript. The tables list all raw data obtained from the analyses of experimental samples. Data are organized by figure and, for each figure, by experimental group.

RAW DATA FIG. 1

Fig.1a

	#	Gcase activity (% Ctr)
**Ctr**	1	116.22
	2	95.29
	3	96.92
	4	86.95
	5	108.17
	6	96.44
**Tg**	1	62.19
	2	80.84
	3	48.36
	4	59.64
	5	79.11
	6	42.39
	7	62.77

Fig. 1c

	#	ASYN optical density (% Ctr)
**Ctr**	1	96.19
	2	99.26
	3	88.71
	4	103.17
	5	112.67
**Tg**	1	140.02
	2	144.23
	3	138.19
	4	118.45
	5	104.05

RAW DATA FIG. 2

Fig. 2b

	#	hASYN IR neurons (#)
**Ctr**	1	99
	2	122
	3	89
	4	44
	5	86
**Tg**	1	92
	2	97
	3	101
	4	111
	5	72

Fig. 2d

	#	hASYN intensity
**Ctr**	1	10782.39
	2	16271.30
	3	10558.75
	4	8258.19
	5	11282.30
	6	13024.45
**Tg**	1	13863.46
	2	14243.48
	3	14830.98
	4	11996.64
	5	15678.94
	6	12110.53
	7	10364.17
	8	11625.34

Fig. 2f

	#	Optical Density (%Ctr)
**Ctr**	1	86.90
	2	84.38
	3	71.40
	4	113.39
	5	143.93
**Tg**	1	148.76
	2	142.49
	3	128.64
	4	96.61
	5	79.74

Fig. 2h

	#	Optical Density (%Ctr)
**Ctr**	1	97.26
	2	94.75
	3	101.11
	4	113.83
	5	93.05
**Tg**	1	101.1403
	2	108.3006
	3	112.844
	4	129.7584
	5	116.3135

Fig. 2j

	#	Optical Density (%Ctr)
**Ctr**	1	101.74
	2	93.30
	3	96.15
	4	113.42
	5	95.39
**Tg**	1	88.19
	2	100.79
	3	112.73
	4	130.02
	5	123.67

RAW DATA FIG. 3

Fig. 3b

	#	hASYN intensity
**Ctr**	1	20171.36
	2	18901.03
	3	21285.67
	4	20711.72
**Tg**	1	18808.10
	2	20372.28
	3	18154.13
	4	21096.15

	#	SynO2 intensity
**Ctr**	1	9322.71
	2	11896.42
	3	11134.84
	4	10294.75
**Tg**	1	14281.12
	2	14057.65
	3	12775.06
	4	14569.99

Fig. 3d

	#	hASYN/hASYN PLA dots (#)
**Ctr**	1	21
	2	13
	3	27
	4	41
**Tg**	1	287
	2	184
	3	52
	4	160

Fig. 3f

		h-ASYN positive axons (#)		
	**#**	**Pons**	**MB**	**FB**
**Ctr**	1	59	7	3
	2	52	4	1
	3	48	3	2
	4	49	5	2
	5	71	6	5
	6	87	6	3
**Tg**	1	118	13	6
	2	103	10	9
	3	122	16	9
	4	88	12	6
	5	101	9	7
	6	130	12	11
	7	131	13	10

RAW DATA FIG. 4

Fig. 4b

	#	Ox-DHE intensity
**Ctr**	1	4001.22
	2	3242.19
	3	3415.25
	4	4646.93
	5	5573.04
	6	4096.61
**Tg**	1	6847.05
	2	6535.93
	3	6060.03
	4	5104.71
	5	7555.15
	6	4429.31
	7	5210.73
	8	9427.40

Fig. 4d

	#	Ox-DHE intensity Right (contralateral)	Ox-DHE intensity Left (ipsilateral)
**Ctr**	1	7.44	8.46
	2	5.64	7.50
	3	6.23	9.55
	4	6.48	7.15
	5	6.59	8.56
	6	6.30	7.99
**Tg**	1	9.02	12.93
	2	8.21	12.85
	3	10.58	13.51
	4	7.83	10.92
	5	7.92	11.40
	6	7.45	14.62
	7	7.69	10.96
	8	9.73	10.61

Fig. 4f

	#	3-NT/hASYN PLA dots (#)
**Ctr**	1	1482.35
	2	1411.76
	3	529.41
	4	1047.06
**Tg**	1	4470.59
	2	4410
	3	4741.18
	4	6847.06

RAW DATA FIG. 5

Fig. 5b

	#	SOD2 intensity
**Ctr+Empty**	1	7286.01
	2	7403.02
	3	7142.15
	4	6349.65
	5	6718.13
**Ctr+SOD2**	1	8208.80
	2	9919.88
	3	11202.37
	4	7211.22
	5	7743.81
**Tg+Empty**	1	7441.43
	2	7129.53
	3	6950.13
	4	7904.48
	5	6480.36
**Tg+SOD2**	1	7257.92
	2	8421.35
	3	11025.22
	4	10791.74
	5	10180.24

Fig. 5d

	#	ASYN optical density (% Ctr+Empty)
**Ctr+Empty**	1	99.42
	2	118.80
	3	81.78
	4	127.78
	5	72.22
**Ctr+SOD2**	1	108.88
	2	107.71
	3	96.23
	4	122.58
	5	120.57
**Tg+Empty**	1	152.40
	2	111.54
	3	98.78
	4	145.69
	5	98.84
**Tg+SOD2**	1	112.51
	2	109.89
	3	73.03
	4	76.45

RAW DATA FIG. 6

Fig. 6b

	#	hASYN intensity	SynO2 intensity
**Ctr+Empty**	1	49847.94	12125.58
	2	50538.62	11009.58
	3	47793.04	10051.58
	4	45456.15	9825.135
	5	47153.91	11015.3
	6	47231.38	11057.72
**Ctr+SOD2**	1	47455.78	11043.19
	2	50835.42	10511.29
	3	45863.66	10236.39
	4	48254.28	8561.376
	5	44101.72	7984.93
**Tg+Empty**	1	47594.53	14530.82
	2	47335.98	15809.32
	3	49342.90	18212.36
	4	44950.20	16672.65
	5	43165.64	14507.37
**Tg+SOD2**	1	46457.66	10477.42
	2	45109.52	12474.71
	3	45976.21	12700.94
	4	46800.17	11924.93
	5	47525.28	11620.66
	6	49495.47	12558.18

Fig. 6d

	#	hASYN/hASYN PLA dots (#)
**Ctr+Empty**	1	11
	2	176
	3	176
	4	274
	5	241
	6	130
**Ctr+SOD2**	1	375
	2	132
	3	36
	4	208
	5	386
**Tg+Empty**	1	708
	2	1481
	3	419
	4	574
**Tg+SOD2**	1	661
	2	390
	3	133
	4	530
	5	133

Fig. 6f

	#	h-ASYN positive axons (#) Pons	MB
**Ctr+Empty**	1	88	10
	2	95	7
	3	84	6
	4	71	5
	5	81	7
	6	51	4
**Ctr+SOD2**	1	73	4
	2	76	9
	3	75	7
	4	82	9
	5	96	8
**Tg+Empty**	1	121	15
	2	131	17
	3	96	16
	4	90	9
	5	142	19
**Tg+SOD2**	1	89	6
	2	105	10
	3	87	7
	4	83	6
	5	99	8
	6	77	6

RAW DATA FIG. 7

Fig. 7b

	#	3-NT/hASYN PLA dots (#)
**Ctr+Empty**	1	5941.18
	2	7376.47
	3	9505.88
	4	20235.29
	5	16988.24
**Ctr+SOD2**	1	8317.65
	2	12752.94
	3	19200
	4	12047.06
	5	20988.24
**Tg+Empty**	1	17952.94
	2	14782.35
	3	23388.23
	4	29482.35
	5	31788.24
**Tg+SOD2**	1	2741.18
	2	13505.88
	3	8988.23
	4	22258.82
	5	19670.59
	6	12188.24

Fig. 7d

	#	3-NT/NDUFB8 PLA (Area fraction)
**Ctr+Empty**	1	1.87
	2	3.78
	3	4.24
	4	6.69
	5	2.58
**Ctr+SOD2**	1	0.62
	2	0.64
	3	3.30
	4	2.79
	5	3.88
**Tg+Empty**	1	11.98
	2	6.56
	3	14.79
	4	9.81
	5	8.03
**Tg+SOD2**	1	4.28
	2	5.73
	3	4.47
	4	8.66
	5	3.40

Fig. 7e

	#	3-NT/NDUFB8 PLA (Integrated density)
**Ctr+Empty**	1	0.76
	2	677789.35
	3	731340.00
	4	606708.03
	5	311727.72
**Ctr+SOD2**	1	105934.17
	2	97706.48
	3	467107.94
	4	574989.25
	5	701699.33
**Tg+Empty**	1	1874076.47
	2	902564.56
	3	2593253.33
	4	2584213.62
	5	1425872.05
**Tg+SOD2**	1	543085.22
	2	1091525.37
	3	1011562.81
	4	1133859.59
	5	833486.63

RAW DATA SUPPLEMENTARY FIG. 1
Supplementary Fig. 1a

		Cell body	
	**#**	**hASYN intensity**	**SynO2 intensity**
**Ctr**	1	2373.726	254.9
	2	2044.583	169.19
	3	2164.936	194.05
	4	2495.359	475.02
**Tg**	1	2195.475	1156.777
	2	2554.804	780.876
	3	2047.434	499.184
	4	2601.474	1264.959

Supplementary Fig. 1b

		Projections	
	**#**	**hASYN intensity**	**SynO2 intensity**
**Ctr**	1	20897.93	10006.32
	2	19352.64	12397.64
	3	21798.63	11698.19
	4	21297.21	10593.95
**Tg**	1	19247.15	15589.28
	2	20188.14	14776.67
	3	19013.53	12870.75
	4	21928.39	15047.15

RAW DATA SUPPLEMENTARY FIG. 2
Supplementary Fig. 2c and d

	**#**	**hASYN intensity**	**MJFR14-6-4-2 intensity**
**Ctr**	1	30569.73	11543.96
	2	27896.28	10677.59
	3	28270.57	10529.91
	4	24166.31	12954.95
	5	31065.51	8428.741
**Tg**	1	28683.59	14787.46
	2	30054.68	12882.26
	3	27877.29	18310.92
	4	26765.01	17283.85
	5	26106.23	11312.38

RAW DATA SUPPLEMENTARY FIG. 3
Supplementary Fig. 3a

		Cell body	
	#	**hASYN intensity**	**SynO2 intensity**
**Ctr+Empty**	1	13976.8	2105.961
	2	14437.04	1858.652
	3	13589.17	2442.765
	4	13141.31	1490.729
	5	15009.88	445.7987
	6	13657.38	1113.253
**Ctr+SOD2**	1	13598.02	431.1218
	2	14331.61	1750.571
	3	12909.55	1415.211
	4	13380.03	1340.92
	5	12794.21	438.9578
**Tg+Empty**	1	13969.81	5161.701
	2	13122.16	5746.425
	3	13769.83	3987.105
	4	13313.46	3672.579
	5	12626.67	6255.899
**Tg+SOD2**	1	12524.03	1858.548
	2	12847.71	1262.69
	3	12249.68	1674.431
	4	13432.06	1588.359
	5	13783.8	835.9891
	6	14018.87	1476.157

Supplementary Fig. 3b

	#	Projections	
		**hASYN intensity**	**SynO2 intensity**
**Ctr+Empty**	1	50147.84	12369.15
	2	50693.26	11523.65
	3	47903.42	10143.73
	4	45663.21	10239.56
	5	47496.39	11605.33
	6	47791.23	11298.29
**Ctr+SOD2**	1	48512.39	11598.36
	2	51004.8	11824.65
	3	46112.4	11024.79
	4	48504.77	8867.954
	5	44298.64	8236.802
**Tg+Empty**	1	48623.22	15669.15
	2	47457.63	16987.45
	3	49660.14	19036.54
	4	45107.52	17604.33
	5	43251.89	14606.33
**Tg+SOD2**	1	46784.13	10977.45
	2	45306.67	12803.23
	3	46172.89	12996.35
	4	47025.95	12157.55
	5	47661.35	12038.7
	6	49783.15	12947.26

RAW DATA SUPPLEMENTARY FIG. 4
Supplementary Fig. 4b

	#	3-NT/NDUFS3 PLA (Area fraction)
Ctr+Empty	1	7.28
	2	7.42
	3	5.69
	4	1.21
	5	1.44
	6	5.16
Ctr+SOD2	1	9.93
	2	2.18
	3	4.65
	4	5.86
	5	2.78
Tg+Empty	1	23.52
	2	17.99
	3	12.42
	4	14.42
	5	32.46
Tg+SOD2	1	7.55
	2	7.56
	3	8.01
	4	7.65
	5	8.98

RAW DATA SUPPLEMENTARY FIG. 6
Supplementary Fig. 6a

		GCase activity (nmol/hr/mg of protein)	
	#	**Male**	**Female**
**Ctr**	1	24.98	18.69
	2	20.48	23.25
	3	20.83	20.73
**Tg**	1	13.37	12.82
	2	17.38	17
	3	10.39	9.11
	4	13.49	

Supplementary Fig. 6b

h-ASYN positive axons (#)											
**Pons**				**MB**				**FB**			
**Male**		**Female**		**Male**		**Female**		**Male**		**Female**	
**Ctr**	**Tg**	**Ctr**	**Tg**	**Ctr**	**Tg**	**Ctr**	**Tg**	**Ctr**	**Tg**	**Ctr**	**Tg**
48	105	53	88	3	13	5	12	2	13	4	6
49	131	49	101	5	13	5	9	3	10	2	7
87	116	47	94	9	10	4	11	6	10	3	10
74	130	67	103	6	12	3	10	1	11	6	9
	118	71	122		13	6	16		6	5	9
